# Retinal Image Dataset of Infants and Retinopathy of Prematurity

**DOI:** 10.1038/s41597-024-03409-7

**Published:** 2024-07-23

**Authors:** Juraj Timkovič, Jana Nowaková, Jan Kubíček, Martin Hasal, Alice Varyšová, Lukáš Kolarčík, Kristýna Maršolková, Martin Augustynek, Václav Snášel

**Affiliations:** 1 University Hospital Ostrava, Clinic of Ophthalmology, Ostrava, 708 52 Czech Republic; 2 University of Ostrava, Faculty of Medicine, Department of Craniofacial Surgery, Ostrava, 703 00 Czech Republic; 3 VSB-Technical University of Ostrava, Faculty of Electrical Engineering and Computer Science, Department of Computer Science, Ostrava, 708 00 Czech Republic; 4 VSB-Technical University of Ostrava, Faculty of Electrical Engineering and Computer Science, Department of Cybernetics and Biomedical Engineering, Ostrava, 708 00 Czech Republic

**Keywords:** Anatomy, Medical imaging, Retinopathy of prematurity

## Abstract

Retinopathy of prematurity (ROP) represents a vasoproliferative disease, especially in newborns and infants, which can potentially affect and damage the vision. Despite recent advances in neonatal care and medical guidelines, ROP still remains one of the leading causes of worldwide childhood blindness. The paper presents a unique dataset of 6,004 retinal images of 188 newborns, most of whom are premature infants. The dataset is accompanied by the anonymized patients’ information from the ROP screening acquired at the University Hospital Ostrava, Czech Republic. Three digital retinal imaging camera systems are used in the study: Clarity RetCam 3, Natus RetCam Envision, and Phoenix ICON. The study is enriched by the software tool ReLeSeT which is aimed at automatic retinal lesion segmentation and extraction from retinal images. Consequently, this tool enables computing geometric and intensity features of retinal lesions. Also, we publish a set of pre-processing tools for feature boosting of retinal lesions and retinal blood vessels for building classification and segmentation models in ROP analysis.

## Background & Summary

Retinopathy of Prematurity (ROP) is a disease of the immature retina caused by the disruption of a newly-formed retinal blood vessels’ physiological development. ROP is the most common cause of severe visual impairment or blindness in children in developed countries. From the last known epidemiologic data, it is indicated that the female gender, gestational age, and birth weight are the influencing factors^1^; according to other studies, the male gender is the factor influencing the ROP severity^2^. To approximate the ROP screening and stage of the ROP, which is monitored as part of the incidence assessment in selected countries classified is listed in Table 1.

**Table 1:** Incidence of ROP according to monitored ROP stage in selected countries^1,2,55–59^.

Country	Stage of the monitored ROP	Incidence
China	Stage 3	1.3–13.1%
Singapore	Stage 3 or more	8.0%
Argentina	Treatable ROP	13.4%
Brazil	Treatable ROP (CRYO-ROP/ETROP)	3.4–5.9%
Colombia	Treatable ROP (CRYO-ROP)	8%
India	Treatable ROP (ETROP/CRYO-ROP)	10.2–11.0%
Kuwait	Treatable ROP	7.8%
Pakistan	Treatable ROP (CRYO-ROP)	20.6%
Saudi Arabia	Treatable ROP	1.2-6.4%
South Africa	Treatable ROP (CRYO-ROP)	4.3%
Vietnam	Treatable ROP (CRYO-ROP)	9.3%
Bangladesh	ROP in all stages	4.4%
Iran	ROP in all stages	8.5%
USA	ROP in all stages	19.88%
Canada	Severe ROP	12.7%
Austria	without definition	16.0%
Belgium	without definition	19.8–15.5%
Germany	without definition	14.8-19.5%
Lithuania	without definition	4.2%
United Kingdom	without definition	5.2%

The regular posterior segment of the eye examinations by an ophthalmologist, so-called ROP screenings, are necessary for all prematurely born infants. It represents a highly specialized area of pediatric ophthalmologic care requiring advanced erudition of an ophthalmologist and the acquisition of specialized equipment - an indirect ophthalmoscope and preferably a digital imaging device with the ability to acquire and evaluate images of the posterior segment of the eye of prematurely born infants over time. As the ROP screening is a specialized area with results in digital images, it is a typical problem where there is room for computer-aided development.

Computer-aided methods are a gateway for extending medical care to remote parts of the world where experienced medical staff are unavailable. The computer-aided diagnostic tools can be useful for initial diagnostics (e.g., triage) and provide inputs for subsequent treatment by expert specialists. Alternatively, in simpler cases, they can speed up the medical processes to ensure health care for more patients. It is necessary to achieve years of experience and available datasets to develop classic or computer-aided screening methods. Also, the waiting times for some examinations and subsequent evaluations can be too long. They can cause the late detection of diseases, which can be associated with high morbidity.

It is necessary to have hands-on examples and experiences for the development of computer-aided methods and, generally, for knowledge widening. These examples and experiences, in the case of automatic processing, can be considered labeled data. In the presented work, retinal image data labeled with patient data will be discussed.

Many studies and huge free available datasets can be found for the retinopathy of adults^3^ very often connected with Diabetes Mellitus or for other parts of the eyes^4^. Unfortunately, the situation of infants’ retina images or direct ROP datasets differs. Over the years, few studies and datasets were published devoted to the problematics of infants and prematurely born infants that are treated for the development of retinal disorders, including ROP. The following paragraph and Table 2 summarizes the survey of the reported infants’ retina image datasets and applied methods to these datasets for processing, analysis, and computer-aided medicine.

**Table 2:** The database and dataset review.

Dataset	Patients	Cases	Images	Labels	Availability
USA–iROP^5,6^	971	—	5,561	normal/plus/pre-plus (labeled by 3 experts)	not available
USA–1^8^ (Ataer-Cansizoglu *et al*.)	—	—	77	normal/plus/pre-plus (labeled by 3 experts)	not available
Canada^9^ (Worrall *et al*.)	35	347	1,459	per-exam (plus/no-plus), per-eye (plus/no-plus) (per-image label is not available)	not available
UK–London^9^ (Worrall *et al*.)	—	—	106	plus/no-plus (labeled by 4 experts)	not available
USA–2^10^ part of iROP (Yildiz *et al*.)	—	—	5,512	normal/plus/pre-plus	not available
USA–3^11^ (Ding *et al*.)	—	—	2,759	stage 1/stage 2/stage 3	not available
Germany^12^ (Walz *et al*.)	90	—	no images	—	on demand
South Korea^13^ (Hong *et al*.)	181,582	—	no images	—	on demand
China - Chengdu^14^ (Wang *et al*.)	1,272	3,722	20,795	normal/minor ROP/severe ROP	not available
USA–4^15^ (Chiang *et al*.)	67	—	248 eyes	—	not available
USA–5^10,16^ (Tian *et al*.)	—	—	100	normal/plus/pre-plus (labeled by 13 experts)	not available
China - Wuchan^17^ (Tong *et al*.)	—	—	38,895 (36,231)	classification (normal/mild/semi-urgent/urgent), identification (demarcation line ridge/ridge with extra retinal fibrovascular involvement/subtotal retinal detachment/total retinal detachment) (labeled by 13 experts)	on demand
China - Shenzhen^18^ (Zhang)	—	—	20,822	disease (8,244)/normal (11,298)/unqualified (1,280)	original not available
Taiwan and Japan^20^ (Huang *et al*.)	210	—	6,500	normal/stage 1/stage 2/stage 3 (labeled by 3 experts)	on demand
India^21^ (Bhatkalkar *et al*.)	—	—	6,043	—	not available
USA–SUNDROP^60,61^	1,570	6,310	79,807	no/mild ROP/“more than mild ROP” (MTMROP)/TR-ROP (labeled by 1 expert)	not available
USA + Canada–e-ROP^23,62^	1,257	4,113	—	demarcation line (stage 1), a ridge (stage 2), extraretinal neovascularization (stage 3), or retinal detachment (stage 4) (labeled by 2 experts)	not available
Czech - Ostrava^40–42^ (Timkovič *et al*.)	188	484	6,004	normal/14 other diagnosis and normal/plus/pre-plus	free

Note: the names listed in the tables must not be the authors of the dataset, but are the authors of the article, where the available information about dataset was found.

A dataset called iROP from the USA (USA–iROP) was published in 2014^5,6^ and provides 5,561 images, but unfortunately it is not available for the professional community. It is assumed, that nowadays the dataset is bigger, but unfortunately, the information is not available. The automatic system for plus disease diagnosis (for a better understanding of plus diseases, see Table 3) and the prediction of features built on deep learning algorithms with a result of clear biomarkers indicating clinical variables^7^ is presented on the data. In 2015 the computer-based image analysis system was published for plus disease classification and analysis. The system is designed to classify the image as plus, pre-plus and normal. Three experts independently classified the images with very comparable results. The system performs with 95% accuracy and is based on the dataset which is designated as the USA–1 dataset. The proposed system is based on the extraction of 11 features (e.g., tortuosity, dilatation etc.) divided into segment-based and point-based features and computed separately for arteries and veins, followed by the Gaussian Mixture Models for each feature to model the vessels. The study uses 77 images^8^. Canadian and London (UK–London) datasets are used to train and evaluate the ROP detection system based on Convolutional Neural Networks (CNN). Fine-tuned and pre-trained GoogLeNet is designed to create a Bayesian posterior over the disease presence and other CNN for a feature map visualization of patologies^9^. Also, parts of the iROP (USA–2) dataset and the USA–3 are used with CNN and feature extraction approach^10,11^. The German ROP register provides data from 90 patients^12^, but no image data is available, just like the South Korean database with 181,582 patients^13^ without images. Deep Neural Networks (DNN) Id-Net (for classification/identification of ROP or normal findings) and Gr-Net (for grading - Minor or Severe ROP grade) are the building blocks of the DeepROP system^14^ based on the China - Chengdu dataset. Another USA–4 dataset consisting of images from 248 eyes (from 67 patients) is presented in^15^. Tian *et al*. present a generative model combining the class and comparison of the label generation severity score on a dataset of 100 images (USA–5)^16^. A 101-layer CNN ResNet (pre-trained) and Faster-RCNN (pre-trained) are tuned on the China - Wuchan dataset^17^ for classification and identification. Another Chinese dataset, the China - Shenzhen dataset containing 20,822 images, is available only in resolution 80 × 60 pixels sorted into three parts - normal, ROP and unqualified (images, which are not useful) and is presented for the usage with the DNN-classifier for normal and ROP images^18^. The same author^19^ published a very similar work, mentioning Telemed-R screening data with 26,424 infant images. The dataset is unavailable and not described deeply; from the context of the article, it is the same dataset as in the previous case. The Taiwanese and Japanese dataset^20^ is used to train five DNN - VGG16, VGG19, MobileNet, InceptionV3 and DenseNet with good results for ROP diagnosis and following the classification of the severity. The location and segmentation of the optic disc with CNN are performed in an Indian dataset of 6,043 images^21^. SUNDROP (Stanford University Network for Diagnosis of ROP)^22^ and e-ROP are datasets mentioned in connection with telemedicine, where the first is one of the big datasets; and the second one is concentrated on infants born 22 to 35. week with lower birth weight than 1251 grams^23^.

**Table 3:** ROP stages and plus-forms description^63,64^.

Stage	Description
Stage 0	Pre-stage 1. There is no noticeable line of demarcation on the retina, but slightly tortuous retinal blood vessels and suggested connections between arteries and veins are present, and it is not long before the demarcation line actually can appear.
Stage 1	Mildly abnormal retinal blood vessel growth. It is usually resolved spontaneously without consequences and does not require treatment; only the child’s regular check-ups are proposed.
Stage 2	Moderately abnormal retinal blood vessel growth. This stage is characterized by extending the demarcation line and creation of the ridge. It is usually resolved spontaneously without consequences and does not require treatment (but with plus form development, treatment is required^33^); only the child’s regular check-ups are proposed.
Stage 3	Severely abnormal retinal blood vessel growth. The abnormal retinal blood vessels grow toward the center of the eye (threshold) instead of following their normal growth pattern along the retina’s surface. This stage is characterized by extraretinal fibrovascular proliferation. It could be resolved spontaneously without consequences, but with plus form development, treatment is required.
Stage 4	Partial retinal detachment. This stage is characterized by traction of the fibrovascular complex of the retina which cause its partial detachment. Tractional retinal detachments can be divided as extrafoveal - Stage 4 A and foveal - Stage 4B. Treatment (pars plana vitrectomy) is required.
Stage 5	Complete retinal detachment. This stage is usually characterized by irreversible visual loss. Treatment (pars plana vitrectomy) is required.
Plus form	
Normal	Retinal blood vessels are without any changes.
Pre-plus	Intermediate stage to the normal vessels and plus-form. The pre-plus form could develop into a plus disease.
Plus	An abnormal tortuosity and dilatation of the retinal blood vessels are presented.

All the aforementioned facts, combined with the lack of free image datasets, are motivation to prepare and present the free, available, and hereafter updated dataset of infants (most of them prematurely born). The proposed dataset provides images with patient data for the physiological (normal) posterior segment of the eye, ROP in various stages, and other posterior segment diseases. According to the authors, it is also necessary and very useful to provide images of other diseases connected with premature or at-term-born infants and with similar pictorial expressions as ROP. The images are also labeled with normal/plus/pre-plus statements. In the comparison of reviewed and available datasets, the presented dataset is free, huge enough with a good resolution, labeled by two independent ophthalmologic experts with a discussion and consensus in the case of eventual disagreements, and verified for usage in many tasks.

The presented dataset is accompanied by the software application for the retinal lesion segmentation–ReLeSeT. ReLeSeT represents a novel software for retinal lesion analysis. The main aim of ReLeSeT software is to automatically detect the morphological shape of selected lesions and consequent geometrical and intensity features extraction. ReLeSeT is composed of two essential parts, which perform retinal image preprocessing by using Bilateral filtration^24^ and consequently, a selected retinal lesion is automatically tracked by using active contour method without edges^25^. Retinal lesions (most often hemorrhage) represent a significant pathological disorder because they reflect the blood vessel activity of the underlying disease. More neurovascularizations lead to more bleeding lesions on the retina. Segmentation of retinal lesions allows for the automatic identification of geometrical and intensity features of retinal lesions, which is proposed in the mentioned software tool ReLeSeT. Retinal lesion identification has substantial clinical importance in tracking the retinal lesion’s development over time, thus predicting its severity. In the clinical practice of ophthalmology, the features of retinal lesions are compared with the optical disc, which is perceived as a reference point because, in contrast with retinal lesions, the optical disc does not change its geometrical features over time.

The presented dataset is prepared for machine processing; all data analyses and Technical validation were implemented in the Python language using standard libraries for data processing and analysis, such as Pandas, etc.

## Methods

Initially, it is necessary to state that all patients or their legal representatives agreed and signed the informed consent before the examination, approved by the Ethical Committee of the University Hospital Ostrava, Czech Republic, reference number 544/2018, following the Declaration of Helsinki. All the data were collected during the routine examinations, and no data was collected, only targeted for the dataset. The patients or their legal representatives agreed to use audiovisual recordings for scientific and educational purposes, which could be presented and published in professional journals under the journal’s and publisher’s defined licenses. Records are taken only from those parts of the body that are directly related to the treatment or examination. During the presentation, personal information about persons and sensitive personal data or other signs that would lead to a closer identification of the person are not published.

ROP is a disease of the immature retina caused by the disruption of newly-formed retinal blood vessels’ physiological development^26,27^, and, in its classical form, can be divided into five stages of development (Table 3). Stage 0, Stages 1 and 2 usually are resolved spontaneously and do not require treatment; only the child’s regular check-ups are proposed. Stage 2 with plus disease (pre-threshold) and Stage 3 (threshold stage) is highly likely to worsen the disease and requires treatment. Stages 4 and 5 are characterized by partial or complete retinal detachment with all negative clinical consequences; for these stages treatment (pars plana vitrectomy) is required. The plus form (characterized by abnormal tortuosity and dilatation of the retinal blood vessels) is a symptom that can occur in any ROP stage. The plus form draws attention to the possibility of a rapid worsening with a worse response to treatment^27–29^. The occurrence of any stage of plus form (pre-plus, plus) increases the severity of the main disease.

The other diseases observed during the ROP screening and included in the dataset belong to hamartomas, hemorrhage, and hypoplasia n. II and toxoplasma chorioretinitis. Moreover, there patients with physiological findings are included in the dataset.

More information about the dataset and subjects included in the study is provided in Section *Data Records*. Briefly, there are 6,004 images from 188 patients, 96 female and 94 male subjects, recalculated to 3,081 images from female patients and 2,923 from male patients. The mean gestational age is 33. week, the mean birth weight is 2017 grams, and the earliest examinations were performed at 30. week of postconceptual age.

### Data collection

Data are collected during regular examinations at University Hospital Ostrava, Clinic of Ophthalmology in the Czech Republic according to the institution and ethics commitment rules. The examinations are performed on all children born before the 32nd gestational week with a birth weight of less than 1500 grams. However, depending on their current clinical condition, older children with higher birth weights may also be included in the screening program. Typically older children or children with a higher birth weight with an unstable clinical condition, requiring oxygen therapy, among other things, e.g. due to Respiratory Distress Syndrome (RDS). The prevalence of the monitored diagnoses is changed for this reason compared to only prematurely born infants. Screening is performed at regular biweekly intervals until the entire retina is developed and supplied with normal blood vessels. This case is usually in the 40th post-conception week of the child’s age (the sum of the gestational week at birth and the child’s chronological age in the weeks after birth). The inspection intervals may be shortened according to the current clinical condition. The aim is to reveal the first signs of ROP, further monitoring the dynamics of changes over time and, in the event of worsening changes in the retina, early initiation of treatment^27,30–32^. Treatment protocols include either the injection of anti-VEGF drugs into the vitreous space of the eye of a preterm infant or laser photocoagulation of the peripheral avascular part of the retina^27,28,33–36^.

**Fig. 1:**
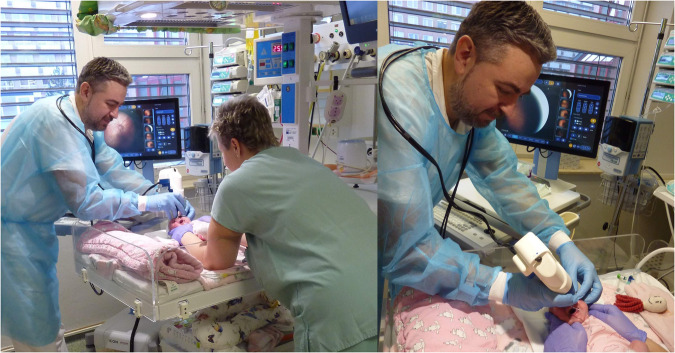
Clinical examination of the prematurely born infant with a digital imaging system (fundus camera) using an eyelid retractor. Note: All the individuals depicted in the image or their legal representatives gave their consent of the image to be openly published.

Most adverse changes occur in the periphery of the retina. For this reason, before the examination, it is first necessary to widen the child’s pupils in both eyes with drops (artificial mydriasis) and ensure that the eyes are opened with an eyelid retractor. ROP screening is performed by an ophthalmologist using indirect ophthalmoscopy and a digital imaging system for the clinical examination of the posterior segment of the eye. The examination is performed while the child is lying down (see Fig. 1).

The resulting quality of the acquired photo documentation is directly proportional to the size (perfection) of the artificial mydriasis (pupil dilation) and the child’s cooperation. Compared to conventional fundus cameras commonly used in collaborating adult patients, the lower resolution of these specialized digital imaging systems is balanced by a wide angle of view, allowing the better visualization and evaluation of changes in the retinal periphery.

Taking images of the posterior segment of the eye is subject to specific conditions. It is a small captured area, a spherical eye environment, limited access to light through the pupil, and a requirement to capture fine details. For objective documentation and diagnostics, specific devices are designed that take these requirements into account. Currently, there are several manufacturers and types of these devices, which, however, are in most cases limited by the need for the cooperation of the examined person and sensing in a sitting position. That is why pediatric ophthalmologists require the construction of a specific device^30–32^. With the growing emphasis on telemedicine services, the development of automation of diagnostics and the growth of pediatric patients with posterior segment disability, and the demands on affordable medical technology, which will enable sufficiently high-quality retinal imaging, are increasing. Clarity RetCam 3, Natus RetCam Envision and Phoenix ICON are used for our purpose.

### Devices description

Clarity RetCam 3 is a widescreen digital imaging system designed with the specific needs of pediatric ophthalmology in mind. It allows taking digital images and short video sequences from retinal examinations for immediate evaluation, objectively comparing findings over time and data sharing. Depending on the lens used, images can be taken at an angle of up to 130 (used in the data collection). Thus, the area shown is significantly larger than binocular indirect ophthalmoscopy (usually a 30-degree view of the retina using a 28D lens and a working distance of 20 mm from the eye’s surface). Fully integrated design and batteries allow the device’s completely independent operation and mobility. Clarity RetCam 3 provides image output with a resolution of 640 × 480 pixels. The lighting is provided by an external halogen light source of 100–6000 lux. The camera is equipped with motorized manual focus (Fig. 1).

Part of the data was collected using the Natus RetCam Envision, one of the Clarity RetCam 3 followers with some new features and improvements. The manufacturer declares that there are five main differences. The image resolution is 1440 × 1080 pixels, so it is 2.25 ×  higher than in Clarity RetCam 3. The new device provides a 130 field of view with one lens. There are two detachable lenses, the first with a 130 view and the second for portrait mode. Detachable lenses are prepared for high-level disinfection. Bi-directional DICOM enables the automatic uploading of the patients’ data to shorten the time; it is not necessary to enter the data manually. When the examination is finished, data are stored for long-term archiving. The other differences are only in the equipment ergonomics.

**Fig. 2:**
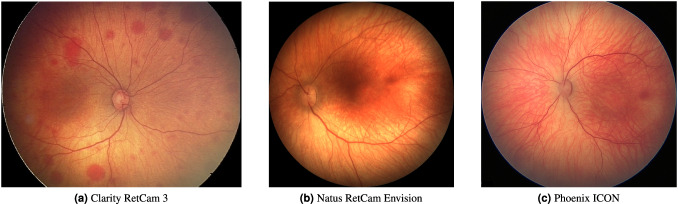
Examples of fundus retinal images taken by Clarity RetCam 3, Natus RetCam Envision and Phoenix ICON.

The rest of the data was obtained using the Phoenix ICON system. During the Phoenix ICON system development, the designers tried to deal mainly with the technical solution of lighting to obtain a sufficiently contrasting image with high resolution (1240 × 1240 pixels), even in the case of imaging highly pigmented parts of the retina. Phoenix ICON uses a sensor that makes it possible to obtain a quality image at a lower light load of the examined child with a 100 field of view with one lens. Compared to Clarity RetCam 3, the handpiece of the Phoenix ICON camera is significantly (30% according to the Phoenix Technology Group) smaller and lighter, and the integrated annular luminaire design (Phoenix Direct Illumination) allows for sufficient image quality with less mydriasis quality (with pupil enlargement from 5.6 mm). The ergonomics of the Phoenix ICON handpiece, the absence of a heavy rigid fiber optic cable, and the ability to zoom in real-time without changing lenses also positively affect the resulting image quality. A comparison of the three mentioned digital imaging systems is shown in (Table 4) and for clarity, the example taken by the aforementioned fundus cameras is depicted in Fig. 2.

**Table 4:** Imaging devices parameters comparison.

	Clarity RetCam 3	Natus RetCam Envision	Phoenix ICON
Illumination	Direct	Direct	Direct
Light source	white light source outside	white light source outside	interchangeable LED light
for fundus for fundus Light source for IVFA	hand piece (wired by fiber optic cable) light source outside hand piece (wired by fiber optic cable)	handpiece (wired by fiber optic cable) light source outside hand piece (wired by fiber optic cable)	module in hand piece interchangeable blue LED light module in hand piece
Real Time Zoom	no	no	yes
Camera gain	no	no	adjustable (0-48 dB)
Field of view	130°, 120°, 80°, 30° changeable lenses	130 field view with 1 lens	100 field view with 1 lens
Sensor	IDS – Single chip camera	IDS – Single chip camera	3MPx
Image resolution	640 × 480 pixels	1440 × 1080	1240 × 1240 pixels
Image formats	MLX (proprietary), DICOM, PNG, JPG	MLX (proprietary), DICOM, bi-directional DICOM, PNG, JPG	8MB Raw (TIFF), JPEG (compressed), DICOM
Video streaming	14 fps	14 fps	30 fps

The next part of the paper is devoted to the software application, which has been developed above the presented dataset and will be described later in Section *Data Records*.

### Retinal lesions segmentation tool–ReLeSeT

**Fig. 3:**
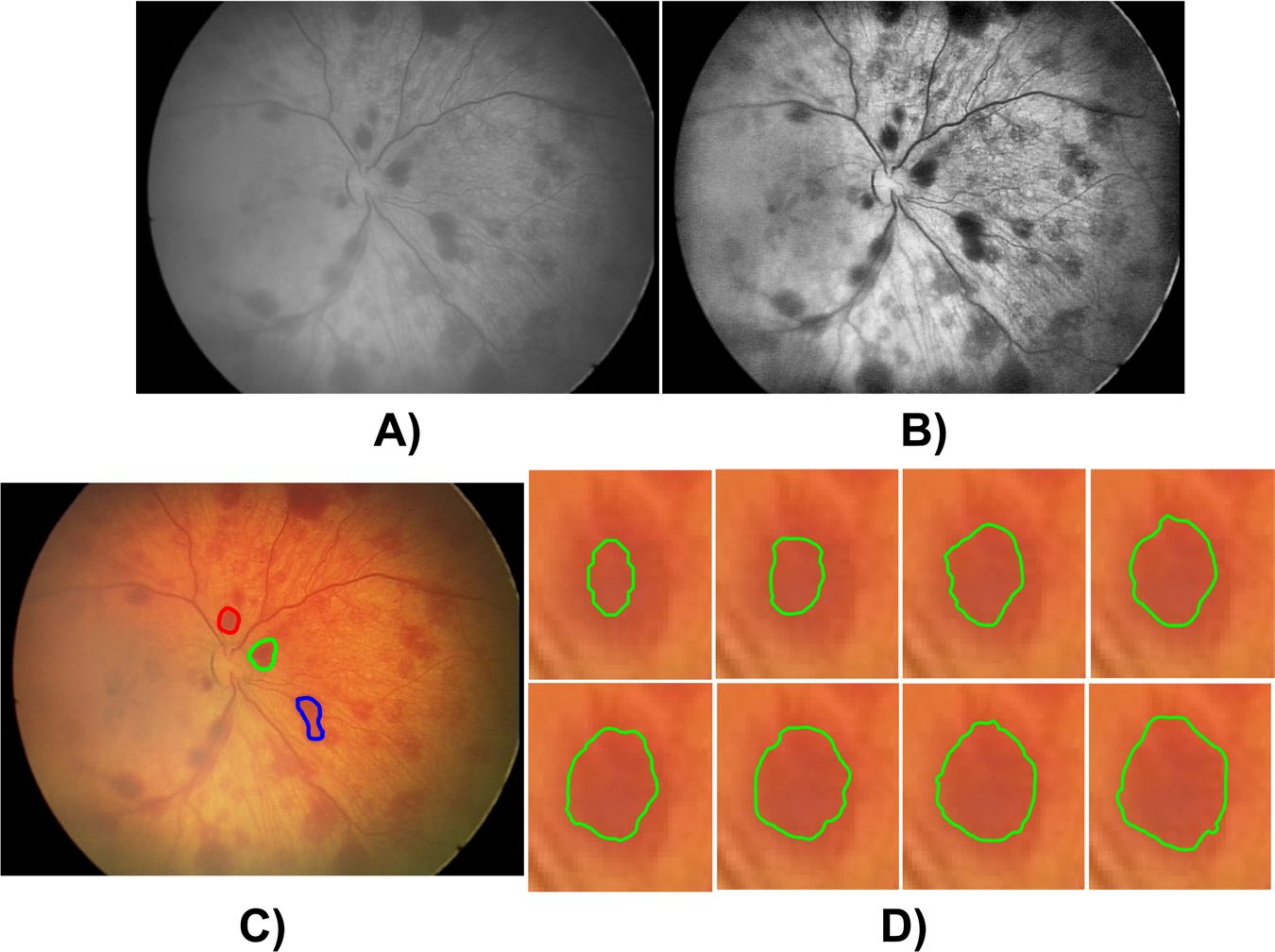
A process of the retinal lesions identification: (**A**) extraction of red and green channel, (**B**) image preprocessing, including adaptive histogram equalization and Bilateral filtering, (**C**) segmentation of retinal lesions based on the active contour model (400 iterations), and (**D**) gradual evolution of retinal lesions segmentation.

This section introduces the software tools intended for the processing and analysis of retinal lesions (most often haemorrhages), which are published together with the retinal image database. The proposed tool can be used for any retinal disease linked with retinal lesions. Thus, segmentation analysis can be generally applied on any type of retinal lesion. The proposed software model (see Fig. 3) includes three parts, which completely consist of the model for retina lesion identification, modeling, and feature extraction. After loading the input retinal image record, image preprocessing is applied (Equation (1)), the retinal lesion is identified based on the active contour model without edges^25^. Lastly, a set of geometrical features is computed for the quantification and objectivization of selected retinal lesions.

In a recent state-of-the-art, there are just a few studies focused on image analysis in retinal lesion segmentation for infants^37,38^. Study^37^ uses a RetCam dataset of 1543 retinal images acquired from 847 newborns and proposes U-net-based architecture for automatic segmentation of retinal blood vessels, optical disc, and retinal lesions. The published deep learning-based approach was designed for Clarity RetCam 3 images only; thus, it is questionable for use with various retinal imaging devices such as Phoenix ICON or Natus RetCam Envision. The proposed approach from the presented study performs segmentation for the retinal lesions from all the mentioned imaging systems. In contrast, the presented study is aimed at individual segmentation of retinal lesions of interest. In our case, a segmentation approach is submitted with geometrical and intensity feature extraction for various retinal images, including Clarity RetCam 3, Natus RetCam Envision, and Phoenix ICON. Thus, ReLeSet should be robust for retinal lesion segmentation from various retinal images.

### Software tools for retinal lesions segmentation

The consequent procedure aims to smooth the retinal image and reduce image noise. This task uses a combination of adaptive image histogram equalization and a Bilateral^24^ filter. This smoothing procedure prevents intensity peaks and image noise inside the retina lesions, thus resulting in better segmentation performance. Assuming the retinal images are in RGB (8-bit) format, represented by a three-level multidimensional matrix, it was experimentally found that the green and red channel combination best reflects the manifestation of retinal lesions. Therefore, a combination of these two channels for further processing is used. A bilateral filter represents a non-linear, edge-preserving smoothing filter aimed at smearing out intensity variability within retinal lesions and respecting image edges at the same time. The mentioned filter^24^ is defined by the following formulation

1 $${I}^{filt}\left(x\right)=\frac{1}{{W}_{p}}\sum _{{x}_{i}\in \Omega }I\left({x}_{i}\right){f}_{i}\left(\left\Vert I\left({x}_{i}\right)-I\left(x\right)\right\Vert \right){g}_{s}\left(\left\Vert \left({x}_{i}\right)-\left(x\right)\right\Vert \right),$$

where *W*_*P*_ represents a normalization term with the definition

2 $${W}_{p}=\sum _{{x}_{i}\in \Omega }{f}_{i}\left(\left\Vert I\left({x}_{i}\right)-I\left(x\right)\right\Vert \right){g}_{s}\left(\left\Vert \left({x}_{i}\right)-\left(x\right)\right\Vert \right).$$

In this formulation, *I*^*filt*^ stands for the filtered retinal image, *I* is the original retinal image with the coordinates *x*, centered in the image domain, where ${x}_{i}\in \Omega$ stands for another pixel. Ω represents a window, which is centered in *x* and *x*_*i*_ is another pixel with index *i*, belonging to Ω. The range kernel *f*_*i*_ aims to compute smoothing differences of the image intensities, and the spatial kernel *g*_*s*_ is the spatial kernel for smoothing differences in coordinates.

The last and most important procedure for the retinal lesion’s identification is the application of the active contour model, which can be perceived as an automatic, iterative, and regional-based segmentation technique with the user initialization (initial seed point). In the proposed software tool, the concept of an active contour without edges is utilized. The active contour is an iteratively deformable continuous curve, which is based on energetic features and smoothly deforms its shape within a predefined number of iterations. The number of iterations is the parameter which is set by a user. The rest of the procedure works fully automatically without the user’s intervention. The whole description of the active contour’s-based segmentation of retinal lesions is provided in appendix.

The proposed segmentation model allows for the identification of individual retinal lesions based on the closed curve, adopting the geometrical shape of retinal lesions. Lastly, the proposed model enables computing selected geometrical features, quantifying individual retinal lesions. The software can compute the geometrical features: the area, perimeter, major, minor axis length of retinal lesions, and intensity distribution of retinal lesions.

### Evaluation of proposed model

In this section, the proposed model for retinal lesion segmentation is evaluated. The active contour, which represents the main element of the identification procedure, is driven by the number of iterations, which represents a number of discrete steps in which the active contour may change its geometrical features to adapt to the retinal lesions. Here, the segmentation performance testing for 400 iterations is presented. This number appears to be a reliable compromise between the computing segmentation time and segmentation performance for all parts of the tested retinal datasets. When using a lower number of iterations (under segmentation), the active contour is unable to adopt the geometrical features of retinal lesions reliably. On the other hand, when using an excessive number of iterations, it goes to over-segmentation, where the active contours sometimes have the tendency to spread out of the retinal lesion’s area. In order to objectively evaluate the segmentation performance, the proposed software tool is tested against manual segmentation (ground truth data, done by clinical ophthalmological experts). The following parameters, except the well-known ones as sensitivity, specificity, and accuracy, are used for the objective evaluation.

- Mean Squared Error (MSE) is one of the most widespread and frequently used reference-based methods for evaluating segmentation accuracy. Considering a 2D image domain with the coordinates (*i*, *j*) and resolution *M* × *N*, we formulate MSE between a ground truth (X) and segmented image (Y) in the following way
  3 $$MSE\left(X,Y\right)=\frac{1}{MN}\mathop{\sum }\limits_{i=1}^{M}\mathop{\sum }\limits_{j=1}^{N}{\left({X}_{i,j}-{Y}_{i,j}\right)}^{2}.$$
- Correlation coefficient (Corr) measures a linear dependence between a ground truth annotation and respective segmentation results. The correlation coefficient is normed within the range [0;1], where 0 stands for no linear dependence; in contrast, 1 stands for full linear dependence.
- Sørensen–Dice Coefficient (DC) of similarity represents an index, representing a ratio between overlapping and non-overlapping regions, defined as

4 $$DC\left(X,Y\right)=\frac{2\left|X\cap Y\right|}{\left|X\right|+\left|Y\right|}.$$

In the case of the correlation coefficient, DC falls in the range where 0 stands for no agreement, while 1 indicates full agreement with the manual annotation. Parameters (X) and (Y) denote the same images as in the MSE formulation.

As can be assumed, the image preprocessing (smoothing) is a crucial procedure for the segmentation due to removing additive noise and smoothing sudden intensity peaks, testing of the segmentation performance using a Bilateral filter in the contrast of Median filtering^39^ (Tables 5, 6) is performed. The segmentation performance was evaluated against ground truth segmentations based on manual contouring by two independent ophthalmological experts, and their results were averaged.

**Table 5:** Evaluation of retinal lesions segmentation for selected retinal lesions for Median and Bilateral filter for device Clarity RetCam 3.

Clarity RetCam 3				
	Validation (30 retinal lesions)		Test (100 retinal lesions)	
	Median filter (average ± σ)	Bilateral filter (average ± σ)	Median filter (average ± σ)	Bilateral filter (average ± σ)
Mean Squared Error	269.56 ± 11.95	214.12 ± 5.30	271.87 ± 21.64	217.16 ± 4.46
Correlation Coef.	0.86 ± 0.028	0.88 ± 0.032	0.85 ± 0.023	0.88 ± 0.033
Sørensen–Dice Coef.	0.84 ± 0.020	0.86 ± 0.030	0.83 ± 0.023	0.85 ± 0.035

**Table 6:** Evaluation of retinal lesions segmentation for selected retinal lesions for Median and Bilateral filter for device Phoenix ICON.

Phoenix ICON				
	Validation (30 retinal lesions)		Test (100 retinal lesions)	
	Median filter (average ± σ)	Bilateral filter (average ± σ)	Median filter (average ± σ)	Bilateral filter (average ± σ)
Mean Squared Error	388.27 ± 6.82	315.12 ± 9.41	394.28 ± 17.33	320.28 ± 10.83
Correlation Coef.	0.85 ± 0.017	0.87 ± 0.047	0.83 ± 0.025	0.85 ± 0.038
Sørensen–Dice Coef.	0.84 ± 0.018	0.88 ± 0.050	0.83 ± 0.052	0.86 ± 0.047

The hold-out test is provided, where images with retinal lesions were divided into two groups: validation and testing data. There was performed testing for the images with retinal lesions from the system Clarity RetCam 3 and Phoenix ICON (Tables 5, 6) separately. In the validation phase, 30 images with retinal lesions were randomly selected, and in the testing phase, 100 images with retinal lesions were for each imaging alternative. The segmentation performance between the Median and Bilateral filters based on the average value ±σ (standard deviation) was evaluated with the parameters MSE, Correlation coefficient, and Sørensen-Dice Coefficient for both validation and testing results. There were used the following settings for the smoothing filters: the kernel size 5 × 5 for median filter and the settings for Bilateral filter: σ_*r*_ = 3 and σ_*s*_ = 0.1 are used for the testing, where σ_*r*_ stands for the dispersion of *f*_*r*_ kernel and σ_*s*_ = 0.1 represents the dispersion of *g*_*s*_ (see Equation (1). It provides a comparative analysis of the Bilateral and Median filter performance for the retinal images from the Clarity RetCam 3 and ICON Phoenix systems due to their significantly different intensity features of retinal blood vessels, optical disc, and retinal lesions. Since the spatial image formation and intensity features of images from the system Natus RetCam Envision are similar to the Clarity RetCam 3, it was not tested the mentioned performance for the Natus RetCam Envision imaging system.

Judging by the averaged results of individual tests for retinal lesions, it is obvious that there are notable differences in evaluation parameters between the Median and Bilateral filters, where for all the evaluation parameters, the Bilateral filter achieved a better performance in the sense of minimization MSE, and maximization of Correlation coefficient and SÃ¸rensen-Dice Coefficient. Further, there were no notable differences between the validation and testing phases (Tables 5, 6). This approach justifies using a Bilateral filter as a better alternative for retinal lesion analysis in ReLeSeT.

Further, an objective evaluation of the segmentation performance of the proposed segmentation algorithm is provided. For the purpose of the testing, the segmentation of retinal lesions from the Clarity RetCam 3, Natus RetCam Envision, and Phoenix ICON datasets are provided. Eighty images with retinal lesions for testing were randomly selected in total from the imaging systems Clarity RetCam 3 (30 images with retinal lesions), Natus RetCam Envision (30 images with retinal lesions), and Phoenix ICON (20 images with retinal lesions). These images with retinal lesions were consequently labeled by two independent ophthalmologic experts; their results were averaged and taken as ground truth data. Based on the ground truth data, the segmentation performance was evaluated. The averaged results, confidence interval (CI), and standard error (SE) are provided in Table 7 for the parameters sensitivity, specificity, accuracy, MSE, Corr, and DC, which evaluates the level of similarity between the ground truth data and the proposed algorithm. All tests were performed on *α* = 0.05 level of significance.

**Table 7:** Performance evaluation for retinal lesions segmentation, where CI is Confidence Interval and abbreviation SE is used for Standard error.

Parameter	Clarity RetCam 3 (average (CI), SE)	Natus RetCam Envision (average (CI), SE)	Phoenix ICON (average (CI), SE)
Sensitivity	0.88 (0.87, 0.88), 0.0033	0.88 (0.86, 0.89), 0.0048	0.99 (0.98, 0.99), 0.0020
Specificity	0.99 (0.98, 0.99), 0.0016	0.99 (0.98, 0.99), 0.0014	0.99 (0.98, 0.99), 0.0012
Accuracy	0.99 (0.98, 0.99), 0.0016	0.99 (0.98, 0.99), 0.0021	0.99 (0.98, 0.99), 0.0018
Mean Squared Error	229.79 (226.92, 232.65), 1.72	231.42 (229.78, 233.05), 0.95	364.85 (362.77, 366.92), 0.97
Correlation Coef.	0.87 (0.86, 0.87), 0.0015	0.86 (0.85, 0.86), 0.0031	0.84 (0.83, 0.84), 0.0047
Sørensen–Dice Coef.	0.81 (0.80, 0.81), 0.0028	0.81 (0.80, 0.81), 0.0020	0.78 (0.75, 0.80), 0.011

**Fig. 4:**
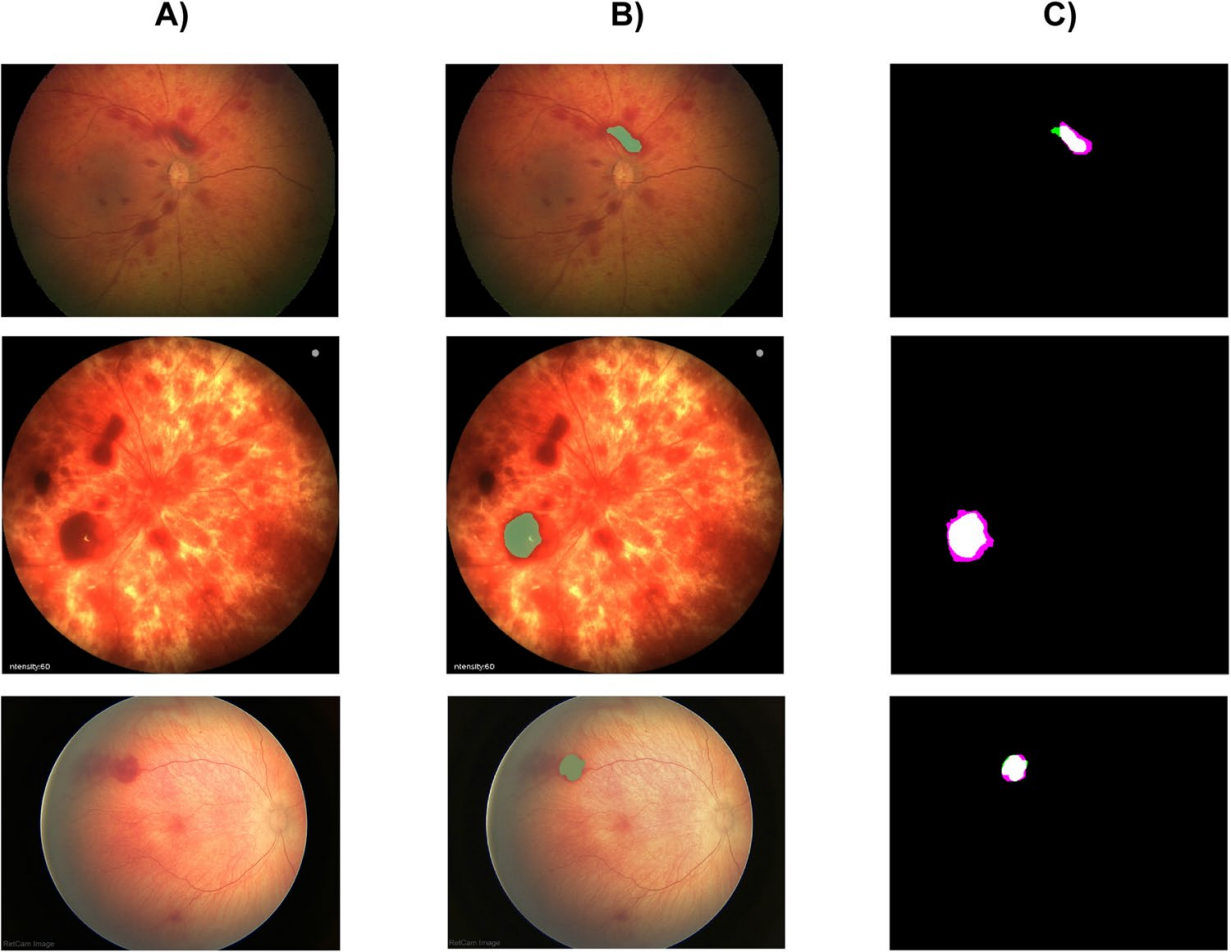
Example of retinal lesions segmentation for Clarity RetCam 3 (up), Phoenix ICON (middle), and RetCam Envision (down): original images (**A**), original images with the segmentation results (**B**), and the overlaying of the segmentation with ground truth, made by neonatal ophthalmologist expert (**C**).

Based on the objective evaluation (Table 7), the differences in the segmentation performance among three retinal imaging systems, Clarity RetCam 3, Natus RetCam Envision, and Phoenix ICON, can be objectively assessed. Based on the provided comparison, it can be concluded that the segmentation for the Clarity RetCam 3 images gives a slightly better performance in the specificity, MSE, Correlation coefficient, and Sørensen-Dice coefficient. On the other hand, only the parameter sensitivity is better for the Phoenix ICON. For better insight of the segmentation performance against the ground truth data, the result of segmentation is depicted in Fig. 4 with the following description–(A) original images, (B) original image with the retinal lesion segmentation, and (C) overlaying the segmentation with the ground truth.

Lastly, the computation costs (Table 8) are evaluated for the same images as the segmentation accuracy. The number of iterations is linked with computation demands. This may be crucial in the case of large lesions, where it is needed to select a higher number of iterations to reliably identify the whole lesion area with higher computational costs. The analysis of computing costs was performed on only the CPU of both PC configurations. To justify the influence of iterations, average computing time, confidence interval, and standard error tested for 50, 100, 200, and 400 iterations for two PC configurations are provided. Table 8 provides the average value for each test, confidence interval, and standard error. All tests were performed on the significance level *α* = 0.05.

1. Intel Core i7-10875H, RAM 16GB DDR4, NVIDIA GeForce RTX 2070 Super Max-Q 8GB,
2. Intel Core(TM) i7-8550U, RAM 8GB DDR4, Intel(R) UHD Graphics 620.

**Table 8:** Average, confidence intervals, and standard error computational costs for two PC configurations for varying number of iterations, where CI is Confidence Interval and abbreviation SE is used for Standard error.

Number of iterations	PC configuration	Computing time (s) (average (CI), SE)	
		Clarity RetCam 3	Phoenix ICON
50	1	3.81 (3.40, 4.21), 0.19	17.14 (15.43, 18.84), 0.83
50	2	4.92 (4.25, 5.58), 0.32	18.55 (16.64, 20.45), 0.93
100	1	12.77 (11.89, 13.64), 0.42	32.79 (30.50, 35.07), 1.11
100	2	18.67 (17.75, 19.58), 0.44	35.51 (34.07, 36.94), 0.70
200	1	21.12 (20.50, 21.73), 0.30	44.41 (43.21, 45.60), 0.58
200	2	28.66 (27.96, 29.35), 0.34	51.19 (49.65, 52.72), 0.74
400	1	266.12 (260.81, 271.42), 2.59	355.22 (351.31, 359.12), 1.90
400	2	354.51 (348.94, 360.07), 2.71	362.11 (356.45, 367.76), 2.76

As discussed in this section, the proposed software ReLeSeT aims to segment selected retinal lesions from variable retinal images automatically. Thus that proves versatile using ReLeSeT for variable retinal image processing as stated in Table 7. On the other hand, some limitations of this segmentation procedure should be considered to provide a complex and objective view of the segmentation procedure. ReLeSeT performs a segmentation procedure of selected lesions, which is further analysed based on the requirements, where physicians are standardly focused on tracking specific lesions. Such procedure is depicted in Fig. 4, where only one selected lesion is extracted and compared against the ground truth data made by clinical neonatal ophthalmologists. On the other hand, utilizing machine learning may bring a new potential to extract all the presented lesions automatically. What is one future direction in this area? The further important issue is the active contour parameter’s setting, especially the number of iterations. Here, all the segmentation tests are provided with 400 iterations, which appear to be a good compromise between the segmentation performance, computing time, and variable manifestation of retinal lesions. Nevertheless, the lesion’s geometrical features, such as size or perimeter, might influence the segmentation performance. In this context, smaller lesions are standardly well segmented with fewer iterations; contrarily, bigger lesions require more iterations. If an improper number of iterations is selected, the performance may be deteriorated by under/over-segmentation. A significant issue is also the intensity profile of retinal lesions. Here, it should be considered that lesions might not have a homogenous intensity profile over the entire lesion’s area, and the lesion’s boundaries do not have to be consistent. These phenomena are partially compensated by image preprocessing. On the other hand, these issues should be considered when processing retinal images from various modalities with different resolutions and intensity profiles. Lastly, the computation costs should be mentioned as a crucial segmentation attribute. In Table 8, significant differences in computing costs may be observable for various numbers of iterations. From this point, the segmentation performance is optimized when using a lower number of iterations. Since the computing costs depend on image resolution because active contours work with image energy, there are notable differences between Clarity RetCam 3 and Phoenix ICON, as these devices have different resolutions. To reduce computing costs, it is advisable to analyze a part of the retinal image, where a retinal lesion of interest is presented instead of the entire image area. The segmentation performance and limitations objectively show the possibility of retinal lesions segmentation and, at the same time, open future possibilities for machine learning for retinal lesions analysis.

### ReLeSeT - instalation and usage

In this part, the proposed segmentation tool for retinal lesion analysis and quantification installation and usage is provided. The implementation enables area extraction, major and minor axis, perimeter, median, and standard deviation of the intensity spectrum for the analyzed retinal lesion. Besides these parameters, the following records are stored: the native image in monochromatic and RGB format, binary model of the retinal lesion, intensity values of the retinal lesion, and preprocessed retinal image. All these records are automatically saved in the variable in the form of a cell array, entitled: *name_Features_results.mat*, where the name denotes the name of the processed retinal image. The entire segmentation procedure is a stand-alone application and run by the file: *ReLeSeT.exe*. It is recommended to have MATLAB Runtime 9.10 (free without installing MATLAB) or higher for this software tool. The parameters for segmentation, including the number of iterations, kernel size of the active contour for retinal lesion segmentation, and the initial active contour parameters – the radius of the initial circle are set in the file: *Segmentation_Parameters.xlsx*. After the segmentation procedure is done, all the results are saved, and the result of the segmentation is visualized over the original retinal image, and the distribution of the intensity spectrum of the segmented lesion is plotted in the form of a histogram.

## Data Records

The files available on Figshare^40^, Kaggle^41^ and GitHub^42^, contains both image folders and data summarization in xlsx format. The release of the published dataset is 2022-11-11. The possible database updates will always be published with the release date.

The published dataset contains 6,004 images from 188 patients. Three approaches for image storage are chosen. The set of identical images is stored in three different root folders. For the first approach, the root folder (‘images’) contains folders with the patient’s ID (identification), and its subfolders contain identification series. The series represents the same patient, with images taken at varying postconceptual ages (different examination dates). Every subfolder contains the real patient’s images taken on the same day from the same patient. Such a division is a user-oriented solution enabling, e.g. educators, quick orientation in the database. The folder root folder (‘images_stack’) contains identical images as in the folder ‘images’, but all images are placed in one folder without other division. The third folder (‘images_stack_without_captions’) is in division similar to (‘images_stack’), the only difference is, that the images are without any incidental caption/label in the image, for more information see Section *Technical Validation*. All the images are in the jpg file format. The jpg files were generated with minimal compression when possible with loss-less compression, considering that one of the devices does not allow the png format. All the images are done in the same format with better compatibility with more devices. Such a solution is more suitable for machine learning algorithms and data processing.

The dataset consisted of posterior segment images when part of the poor quality pictures was deleted, but part was left in the dataset (even with worse quality) for dataset variability. There is a big difference between datasets from older people cooperating during the examination. The infant patients are not cooperating, and it is hard to gain only good images.

All images have a name in a predefined format sequentially describing the following values, where the name in brackets is a shortcut for the given parameters, which are divided by an underscore

**Table:** 

Patient’s ID_sex_gestational age (GA)_birth weight (BW)_postconceptual age (PA)_diagnosis code (DG)_plus-form (PF)_device(D)_serie (S)_image number.jpg.	

The patient information is also summarized in an Excel file (infant_retinal_database_info.xlsx). All information is anonymized and cannot be assigned to a specific patient, even directly by the patient himself.

For instance, the first patient (001_F_GA41_BW2905_PA44_DG2_PF0_RC3_S01_1) is a female (F) with a gestational age (GA) of 41. week, a birth weight (BW) of 2,905 grams, a postconceptual age (PA) of 44. week (all the images were taken at this postconceptual age), diagnosis (DG) 2 – hemorrhage, normal (without plus-form), images taken by Clarity RetCam 3, series (S) n. 1. See Table 10 for diagnosis identification. The image was taken from Clarity RetCam 3 device. Note that the identification of the given series is present in the name of any file. However, postconceptual age is always the same for given series, and it can handle a distinction between series.

### Dataset parameters description

As mentioned, the dataset consists of 6,004 images from 188 patients accompanied by the patients’ information. The variables and their values are described below and summarized in the Table 9. The image dataset is accompanied by the Excel format file (infant_retinal_database_info.xlsx) with all information. The patient and image information is also coded in the image name. Let’s describe the dataset parameters.

**Table 9:** Patients information (variables) summarization, its data type and possible values.

Parameter	Parameter ID	Data type image name	Possible Values	Accompanied in image name by
ID	ID	Integer	—	—
SEX	SEX	Category/Char	F/M	—
GESTATIONAL_AGE	GA	Integer	—	GA
BIRTH_WEIGHT	BW	Integer	—	BW
POSTCONCEPTUAL_AGE	PA	Integer	—	PA
DIAGNOSIS_CODE	DG	Category/Integer	0–13	DG
PLUS_FORM	PF	Integer	0/1/2	PF
DEVICE	D	Integer	1/2/3	D
SERIES_NUMBER	S	Integer	—	S

#### Patient’s ID, sex

The patient’s ID is a unique identifier without any possibility of traceability to a specific patient. The dataset is completely anonymized, and there is no way anybody or even the patient can assign images and data to a specific patient. The ID is an integer, and new data will be added chronologically.

(M). The value is taken from the patient’s material from the maternity hospital.

**Fig. 5:**
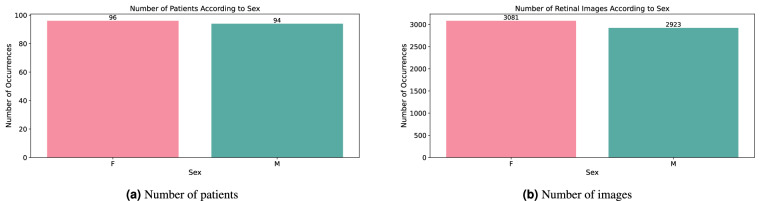
Patients and retinal images distribution according to sex.

The dataset contains images from 188 patients - 94 male and 94 female patients. The dataset is balanced in this respect, and there were no used triage parameters. The retinal images according to the sex are in the ratio 3081:2923 for female, resp. male (Fig. 5).

#### Gestational age

The gestational age is the patient’s age at birth in weeks at the birth of the baby; the value is taken from the patient’s material from the maternity hospital. Gestational age is an integer and is reported in weeks.

**Fig. 6:**
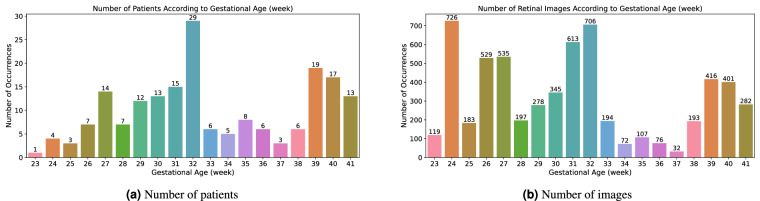
Patients and retinal images distribution according to gestational age in weeks.

The mean gestational age is 33. week, with a standard deviation of 5. The youngest patient was born at 23. week. Contrary, 13 patients have the maximal value of 41. week of gestational age. The distribution of the patients’ number and the retinal images according to the gestational age is depicted in Fig. 6.

#### Birth weight

The birth weight is the patient’s weight at birth; the value is taken from the patient’s material from the maternity hospital.

**Fig. 7:**
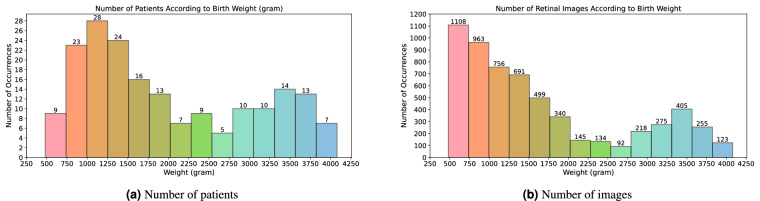
Patients and retinal images distribution according to birth weight in grams.

The mean birth weight is 2017 grams, the standard deviation is 1024 grams, the minimal weight is 480 grams, and the maximal weight is 4080 grams (Fig. 7).

#### Postconceptual age

The postconceptual age is defined as the gestational plus chronological ages. It could also be found in the literature as postmenstrual age^43^. It is an integer-type value and is reported in weeks. The common indicator for the given series of images is the postconceptual age, as all images from the series were taken at the same moment.

**Fig. 8:**
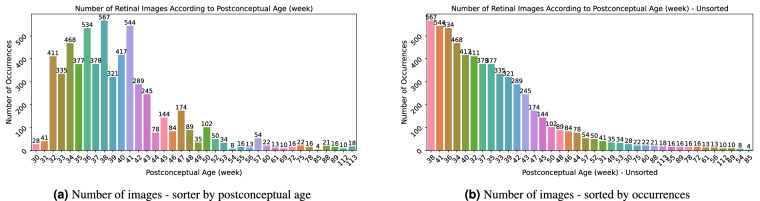
Retinal images distribution according to postconceptual age in weeks.

The earliest examinations were done at 30. week of postconceptual age. At the opposite end of the spectrum, an examination at 113. week was recorded (it is not the first examination of the patient, but the series was done at this age). The distribution of the retinal image number is depicted in Fig. 8.

#### Diagnosis

All defined possible diagnoses are coded according to Table 10. The data is categorical data with an integer type. There are some diagnoses (ROP 4 A, 4B and 5 are not observed in the dataset) with no images; it is not a mistake. The database is prepared for further extension, so it will be useful if the value labels stay the same in the new release. The mentioned diagnoses are considered in the diagnosis list.

**Table 10:** The total number of patients, series and images for given diagnosis.

Diagnosis	Diagnosis code (DG)	Description	Number of patients	Number of series	Number of images
Physiological	0	Physiological finding	135	278	2,980
ROP 0	1	Retinopathy of Prematurity Stage 0	4	5	45
ROP 1	2	Retinopathy of Prematurity Stage 1	12	16	252
ROP 2	3	Retinopathy of Prematurity Stage 2	11	28	458
ROP 3	4	Retinopathy of Prematurity Stage 3	2	4	125
ROP 4 A	5	Retinopathy of Prematurity Stage 4 A	0	0	0
ROP 4B	6	Retinopathy of Prematurity Stage 4B	0	0	0
ROP 5	7	Retinopathy of Prematurity Stage 5	0	0	0
A - ROP	8	Aggressive Retinopathy of Prematurity	1	16	470
St.p. ROP	9	Status post Retinopathy of Prematurity	13	27	379
Hamartomas	10	Retinal astrocytic hamartomas	2	4	72
Haemorrhage	11	Retinal hemorrhages - abnormal bleeding within the delicate retinal blood vessels	35	90	4,061
Hypoplasia n.II	12	Optic Nerve Hypoplasia	2	7	48
Toxoplasma	13	Toxoplasmosis chorioretinitis - infection caused by the Toxoplasma gondii parasite	1	9	114

Diagnosis code (DG) represents inner code of diagnosis for sorting purposes in dataset.

Two ophthalmological experts did the diagnosis and plus-form ascertaining. The cases where the results differed were discussed with the third expert, and a consensus was reached. Only in approximately 4% of the cases was it necessary to request a third expert opinion.

Note that the diagnosis can change over time, i.e., different series can have varying diagnoses from the same patient (but for most patients, the diagnosis stays the same in all series). The image series of the given patient consists of all images obtained from the examination; the diagnosis can be identifiable from the retinal images in the series. As the diagnosis can change over time, the number of patients in Table 10 can not be summed, so the number differs from the number of patients in the dataset.

**Fig. 9:**
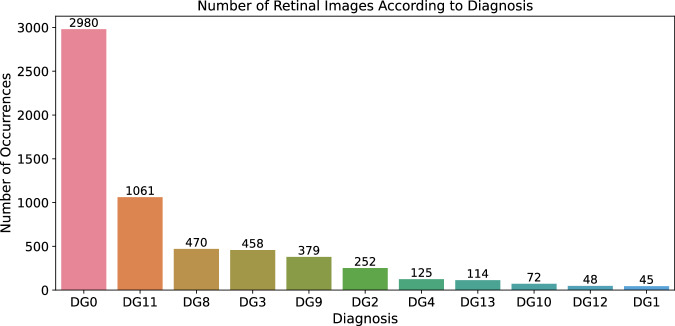
Retinal images distribution according to diagnosis.

The retinal image distribution to the individual diagnoses is depicted in Fig. 9. The distribution of the patients to the diagnoses is not used, as the diagnosis can change over time.

The ratio of physiological images number and the rest of the images is not ideal in some point of view. The dataset is valuable for identifying healthy patients; if we can identify healthy patients, the rest of them can be considered ill. Many illnesses are not identifiable only from the images as they have the same or very similar manifestation on the retina, so even the ability to filter healthy and unhealthy patients is valuable. It could also be used as a learning source for young specialists.

#### Plus form

**Fig. 10:**
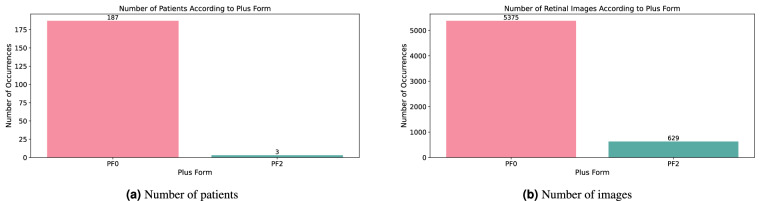
Patients and retinal images distribution according to diagnosed plus form.

The plus form is a symptom that can occur in any ROP stage but only in connection with ROP diagnosis, so the number of occurrences is not high. The variable is an integer type with three possible values: 0, 1, or 2. The number of patients and retinal images with their plus form is depicted in Fig. 10; the pre-plus form is not observed in the dataset. The patients’ division according to the plus form variable could be confusing as both patients with observed non-normal plus form (PF2) were in the beginning diagnosed as patients with normal plus form (PF0), so these two patients are counted twice–as patients with PF0 and PF2. The definition of pre-plus, respectively, the plus form of the disease, is vague^44^, and the patients with pre-plus can be included in the plus-form part. It is also the limitation of all other datasets concerning the pre-plus and plus-form. The incidence of the pre-plus and plus-form is, moreover rare. All the patients with non-normal plus forms (PF1 and PF2) were indicated for the treatment, such as laser or treatment in injection form, whereas the active substance Ranibizumab (known under the trade mark Lucentis) was used.

The plus form ascertaining, the same as the diagnosis, was done by two ophthalmological experts. The cases where the results differed were discussed with the third expert, and a consensus was reached.

#### Device

The devices and their parameters used for the image taking are described in Subsection *Device Description*. The variable is categorical with values of the integer type.

**Fig. 11:**
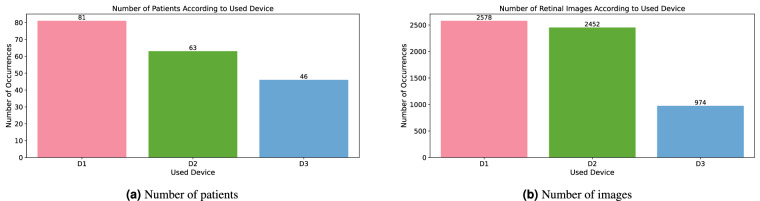
Patients and retinal images distribution according to used device.

All series for one patient were done with the same device. The number of patients and retinal images can be found in Fig. 11.

#### Series number

Variable series could be considered redundant, as the series could be identified from the postconceptual age (reported in weeks). However, in one week, more examinations can be done, so the postconceptual age is not unique for examining the patient, and the variable series has to be added. The variable series is categorical data with an integer type.

**Fig. 12:**
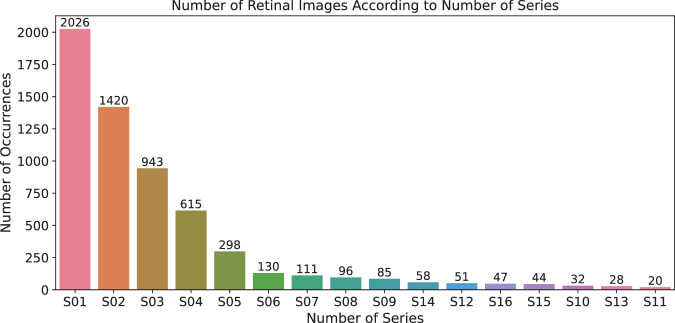
Retinal images distribution according to series number.

Most of the patients (140 patients) were observed more than once, i.e. there are several series of images from multiple visits by some patients; see the number of series in Table 10. Figure 12 shows the number of images in all series sorted by the number of occurrences in the given series.

#### Overview

For better clarity, the dataset variables or parameters, their shape, basic division, and for numerical variables, basic statistical values are provided in Table 11.

**Table 11:** The overview of the variables, its shape and values.

Variable		Number	Division	Number
Patients		188	Female	94
			Male	94
Images		6,004	Female	3,081
			Male	2923
Series (Exams)		484	Female	229
			Male	255
Plus Form	Patients	188^a^	PF0	187
			PF1	0
			PF2	3
	Images	6,004	PF0	5,375
			PF1	0
			PF2	629
	Series (Exams)	484	PF0	463
			PF1	0
			PF2	21
Device	Patients	188	Device 1	81
			Device 2	61
			Device 3	46
	Images	6,004	Device 1	2,578
			Device 2	2452
			Device 3	974
	Series (Exams)	484	Device 1	231
			Device 2	190
			Device 3	63
Variable	Mean	Min	Max	STD
Gestational Age (.week) for patient	33	23	41	5
Birth Weight (gram) for patient	2017	480	4080	1024
Postconceptual Age (.week) for serie (exam)	39	30	113	9

^a^Patients’ division is described in *Plus Form* Subsection.

## Technical Validation

**Fig. 13:**
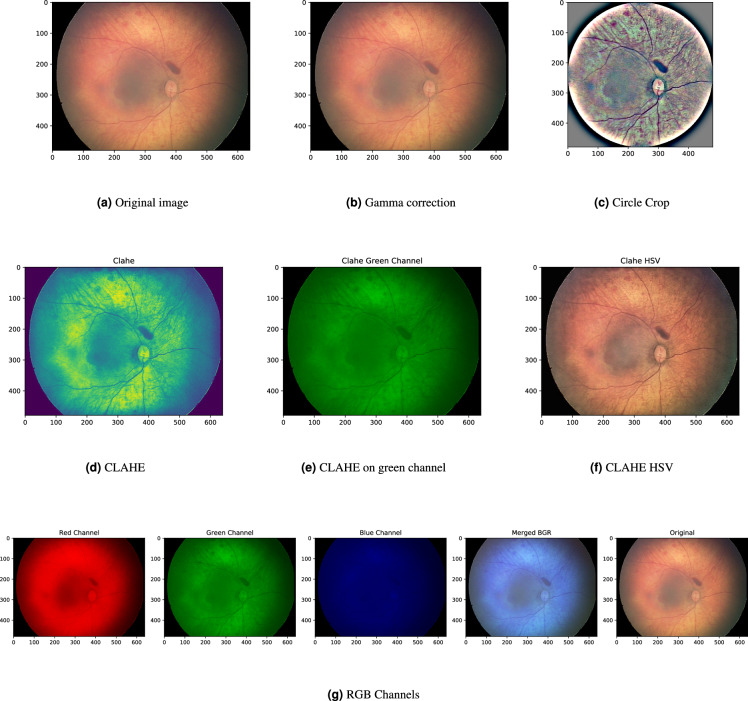
Image enhancements for the random retinal image done by the Device n. 1.

**Fig. 14:**
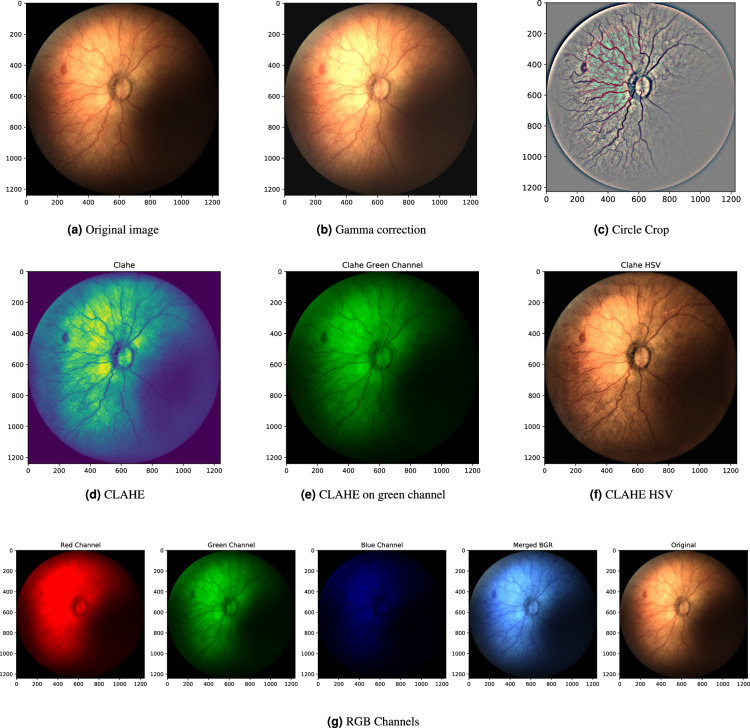
Image enhancements for the random retinal image done by the Device n. 2.

**Fig. 15:**
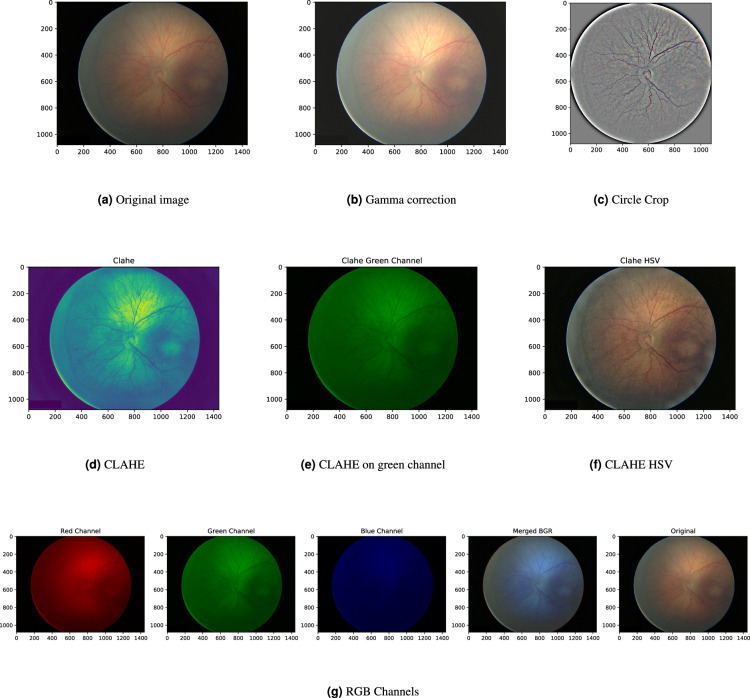
Image enhancements for the random retinal image done by the Device n. 3.

The images and the patient data come from real observations. There were no triage parameters used from any point of view. For the assessment of the image quality and future usage, the basic image enhancement methods for random images from all three devices are presented (see Subsection *Image Enhancement* and Figs. 13–15). A detailed description of the datasets is done in the previous section.

The images of the patient data were created by standardized retinal cameras; see Section *Device Description*. The used devices produce results in standardized jpg format, which is readable by most image reader platforms. Note that retinal cameras print some information, e.g., intensity value, in the black regions at the screen’s corners. Such details are part of snapshot images afterwards. Such captions were removed from the images by replacing the caption with a black rectangle. The camera’s software always manually blacks the outer regions of fundus images. Hence, the replacement does not change the image. The fundus images were never touched during this replacement.

As every image in the dataset is in standardized jpg format, the retinal images can be processed by a lot of image processing software. For instance, the retinal images’ technical validation is demonstrated by using the ReLeSeT (see Section Retinal Lesions Segmentation Tool–ReLeSeT) with the presented retinal images, which is implemented in Matlab Software and furthermore proposed image enhancement methods.

### Image enhancement

Ophthalmologists visually diagnose the structures in retinal images, sometimes such structures are not easy to see by the human eye. In such cases, image enhancement methods make some structures more visible and it helps in computer-aided diagnosis^45–47^. Premature children are not fully cooperating patients and sometimes images are taken when the child is moving. In this case, retinal images are not perfect and image enhancement methods can transform the images into a form which can be used for the establishment of a diagnosis.

The improved (adjusted) digital images can help practitioners or students identify key structures in fundus images, which can lead to more precise diagnoses. Also, the successful application of image enhancement methods provides the technical validation of a dataset for further applications in image processing domains, like machine learning, image enhancement, computer-aided diagnosis, etc. Hence, all images for all three devices were tested by the image enhancement methods. The results, for one randomly selected image from each device are presented in Figs. 13–15(a).

The first tested image enhancement method is gamma correction, i.e., an increase/decrease of the brightness value. The method can be useful as the retinal images of East Asian, Indo-Aryan, Asian, and African Americans are darker, difficult to examine, and even harder to take a picture of the posterior segment of the eye^48^. Figures 13–15(b) shows the application of the brightness enhancement method presented in^49^. It computes the average-per-pixel brightness by a geometric mean, marked as *b*. The authors define the threshold *b*_*min*_ and increase the brightness by a simple linear image transformation if the average image brightness is lower than *b*_*min*_. Alternatively, this transformation can be used to decrease the brightness and standardize the output.

The fixed-aspect transformation method, mostly used as standardized input for machine learning methods, is to crop images from black edges and create a mask in a circle shape with a radius equal to the *min*(*width*/2, *height*/2) of the cropped image. The image within this mask is extracted and has a fixed width = height ratio. It can be resized to any width = height value and does not suffer from aspect ratio deformation. Further, Gaussian Smoothing filtering was applied. It changes the fundus color but enhances the hardly visible structures. The image, after Gaussian Smoothing with a standard deviation along X-axis equal to 15, was added to a cropped image by the weighted sum of the smoothed and cropped images by

5 $$dst=im{g}_{processed}\ast \alpha +im{g}_{smoothed}\ast \beta +\gamma ,$$

with parameters *α* = 5, *β* = −5, and *γ* = 128, which produce good results by internal testing, see Figs. 13–15(c). This transformation results in different colors from the original input, but it significantly enhances all structures. Note, the circle crop cut is used here before Gaussian Smoothing, but Gaussian Smoothing can be used without a circle crop.

The next applied image enhancement method to enhance the retinal structures is the Contrast Limited Adaptive Histogram Equalization (CLAHE) method^50^. CLAHE is a variant of adaptive histogram equalization techniques. Such techniques are used to adjust the global contrast of an image by updating the image histogram’s pixel intensity distribution, i.e., to make the dark portion darker and the bright portion brighter. The goal is to improve the input, i.e., to enhance the local contrast of an image. This property is desirable in infant retinal images as it helps distinguish the structures on retina images (the retina is not fully developed at this age). From a medical point of view, it enhances the structures (e.g. aneurysms), but the veins, lesions, optical disc, and other objects on retinal images can be recognized.

CLAHE can be directly applied to single-channel images like grey-scale or green channel, where each pixel is represented by the intensity value from zero to 255, see Figs. 13–15(d,e). Note, Figs. 13–15(d) is a grayscale image, but in *viridis colormap*, in which structures are even more distinguishable. The application of CLAHE to RGB images is not straightforward due to the presence of three individual channels. The aim is to enhance the brightness, so the image is transferred to HSV channels, where V stands for lightness/value. The CLAHE algorithm is then applied to the V channel, and the image is transformed back from HSV to RGB format. The results are visible on Figs. 13–15(f).

The last and simple image enhancement method is to split images into individual Red, Green, and Blue color (RGB) channels, as structures in fundus images are in higher contrast in the green channel. As a side effect, it also shows potential to use images in deep learning techniques, which benefits from three-channel input^51^. The change of the RGB to BGR spectrum is possible, but it does not provide any improvement of image readability, see Figs. 13–15(g).

All image enhancement methods were implemented in Python 3.9 using the OpenCV 4.6.0 library. This combination can be used for processing images by machine learning methods, and such a dataset is proven to be used in any following applications. A detailed description of the dataset is done in the previous sections.

## Usage Notes

The authors encourage the readers to widen the useful methods for image enhancement^49^ and data analysis applications on the presented dataset; some of the methods can be found on GitHub^45,49,52,53^.

The dataset is prepared for machine processing; all data analyses were implemented in the Python language using standard libraries for data processing and analysis, such as Pandas, etc.

In this study, ReLeSeT software (ReLeSeT can be found on GitHub^54^) is provided, which represents the tools for the automatic processing of retinal lesions from retinal images with the goal of the identification of the selected lesion and further features extraction. ReLeSeT software can be used for all the datasets from this study, including Clarity RetCam 3, Natus RetCam Envision, and Phoenix ICON.

## Appendix

### Local Gaussian Distribution Fitting

It is implemented as an implicit active contour model based on local intensity distribution^25^. To effectively exploit information on local intensities, it is necessary to characterize the distribution of local intensities via the partition of neighborhoods. For each point *x* in the image domain Ω, it is considered a circular neighborhood with a small radius *ρ* which is defined as ${\theta }_{x}\mathop{\Delta }\limits_{=}\left\{y:\left|x-y\right|\le \rho \right\}$. Let ${\left\{{\Omega }_{i}\right\}}_{i=1}^{N}$ denote a set of disjoint image regions, such that $\Omega ={\bigcup }_{i=1}^{N}{\Omega }_{i},{\Omega }_{i}\cap {\Omega }_{j}={\rm{\varnothing }},\forall i\ne j$, where *N* refers to the number of regions. The regions ${\left\{{\Omega }_{i}\right\}}_{i=1}^{N}$ produce the partition of the neighborhood *θ*_*x*_, i.e., ${\{{\Omega }_{i}\cap {\theta }_{x}\}}_{i=1}^{N}$. Now, it is considered the segmentation of this circular neighborhood *θ*_*x*_ based on *maximum a posterior probability* (MAP). Let *p*(*y* ∈ Ω_*i*_ ∩ *θ*_*x*_ | *I*(*y*)) be the *a posteriori* probability of the subregions Ω_2_ ∩ *θ*_*x*_ given the neighborhood gray values *I(y)*.

According to the Bayes rule

6 $$p\left(y{\in \Omega }_{i}\cap {\theta }_{x}| I\left(y\right)\right)=\frac{p\left(I\left(y\right)| y{\in \Omega }_{i}\cap {\theta }_{x}\right)p\left(y{\in \Omega }_{i}\cap {\theta }_{x}\right.}{p\left(I\left(y\right)\right)},$$

where *p*(*I*(*y*)|*y* ∈ Ω_*i*_ ∩ *θ*_*x*_), denoted by *p*_*i*,*x*_(*I*(*y*)) is the probability density in region Ω_*i*_ ∩ *θ*_*x*_ i.e., the gray value distribution within this region. *p*(*y* ∈ Ω_*i*_ ∩ *θ*_*x*_) is the *a priori* probability of the partition Ω_*i*_ ∩ *θ*_*x*_ among all possible partitions of *θ*_*x*_, and *p*(*I*(*y*)) is the a priori probability of gray value *I*(*y*), which is independent of the choice of the region and can therefore be neglected.

Given all partitions are *a priori* equally possible, i.e., *p*(*y* ∈ Ω_*i*_ ∩ *θ*_*x*_) = 1/*N*, the term *p*(*y* ∈ Ω_*i*_ ∩ *θ*_*x*_) can be ignored. Assuming that the pixels within each region are independent, the MAP will be achieved only if the product of *p*_*i*,*x*_(*I*(*y*)) across the regions *θ*_*x*_ is maximized

7 $$\mathop{\prod }\limits_{i=1}^{N}\prod _{y{\in \Omega }_{i}\cap {\theta }_{x}}{p}_{i,x}\left(I\left(y\right)\right).$$

With a logarithm usage, the maximization can be converted to the minimization of the following energy, denoted by ${E}_{x}^{LGDF}$

8 $${E}_{x}^{LGDF}=\mathop{\sum }\limits_{i=1}^{N}\mathop{\int }\limits_{{\Omega }_{i}\cap {\theta }_{x}}-{\log }{p}_{i,x}\left(I\left(y\right)\right){\rm{d}}y.$$

There are various approaches to model the probability densities *p*_*i*,*x*_(*I*(*y*)), including Gaussian density with fixed standard deviation, a full Gaussian density, or a non-parametric Parzen estimator. Most image segmentation methods assume a global model for the probability of each region, i.e., the probability density function only depends on the region but does not change within one region. Consequently, these methods have difficulties in the presence of intensity inhomogeneity. In this paper, it is assumed that the mean and variance of the local Gaussian distribution are spatially varying parameters, i.e.,

9 $${p}_{i,x}\left(I\left(y\right)\right)=\frac{1}{\sqrt{2\pi }{\sigma }_{i}\left(x\right)}{\exp }\left(-\frac{{\left({u}_{i}\left(x\right)-I\left(y\right)\right)}^{2}}{2{\sigma }_{i}{\left(x\right)}^{2}}\right),$$

where (*u*_*i*_(*x*) and *σ*_*i*_(*x*) are local intensity means and standard deviations, respectively. By introducing a weighting function into the function (8), it is defined the following objective functional

10 $${E}_{x}^{LGDF}=\mathop{\sum }\limits_{i=1}^{N}{\int }_{{\Omega }_{i}\cap {\theta }_{x}}-\omega \left(x-y\right){\log }{p}_{i,x}\left(I\left(y\right)\right){\rm{d}}y,$$

where *ω*(*x* − *y*) is a non-negative weighting function such that *ω*(*x* − *y*) = 0 for |*x* − *y*| > *ρ* and ${\int }_{{\theta }_{x}}\omega \left(x-y\right){\rm{d}}y=1$. Although the choice of the weighting function is flexible, it is preferable to use a weighting function *ω*(*x* − *y*) such that larger weights are assigned to the data *I*(*y*) for *y* closer to the center *x* of the neighborhood *θ*_*x*_. The weighting function *ω* is chosen as a truncated Gaussian kernel with a localization property that *ω*(*d*) decreases and approaches zero as |*d*| increases,

11 $$\omega \left(d\right)=\left\{\begin{array}{cc}\frac{1}{a}\exp \left(-\frac{| d{| }^{2}}{2{\sigma }^{2}}\right) & if| d| \le \rho ,\\ 0 & if| d|  > \rho ,\end{array}\right.$$

where *a* is a constant such that ∫*ω*(*d*) = 1 The above objective function ${E}_{x}^{LGDF}$ can be rewritten as

12 $${E}_{x}^{LGDF}=\mathop{\sum }\limits_{i=1}^{N}{\int }_{{\Omega }_{i}}-\omega \left(x-y\right)\log {p}_{i,x}\left(I\left(y\right)\right){\rm{d}}y,$$

due to the localization property of the weighting function, namely *ω*(*x* − *y*) = 0 for *y* ∉ *θ*_*x*_. The ultimate goal is to minimize ${E}_{x}^{LGDF}$ for all the center points *x* in the image domain Ω, which directs us to define the following double integral energy functional

13 $${E}^{LGDF}={\int }_{\Omega }{E}_{x}^{LGDF}{\rm{d}}x={\int }_{\Omega }\left(\mathop{\sum }\limits_{i=1}^{N}{\int }_{{\Omega }_{i}}-\omega \left(x-y\right)\log {p}_{i,x}\left(I\left(y\right)\right){\rm{d}}y\right){\rm{d}}x.$$

### Level Set Formulation

It is assumed that the image domain can be partitioned into two regions: foreground and background, denoted by Ω_1_ and Ω_2_, respectively. These two regions can be represented as the regions outside and inside the zero-level set of Φ, i.e., Ω_1_ = {Φ > 0} and Ω_2_ = {Φ < 0}. Using Heaviside function *H*, the energy ${E}_{x}^{LGDF}$ in Eq. (12) can be expressed as an energy in terms of Φ, *u*_*i*_ and ${\sigma }_{i}^{2}$

14 $$\begin{array}{lll}{E}_{x}^{LGDF} & = & (\Phi ,{u}_{1}(x),{u}_{2}(x),{\sigma }_{1}{(x)}^{2},{\sigma }_{2}{(x)}^{2})\\  & = & -\int \omega (x-y){\log }{p}_{1,x}(I(y)){M}_{1}(\Phi (y)){\rm{d}}y\\  &  & -\int \omega (x-y){\log }{p}_{2,x}(I(y)){M}_{2}(\Phi (y)){\rm{d}}y,\end{array}$$

where *M*_1_(Φ(*y*)) = *H*(Φ(*y*)) and *M*_2_(Φ(*y*)) = 1 − *H*(Φ(*y*)). Thus, the energy *E*^*LGDF*^ in Eq. (13) can be rewritten as

15 $${E}^{LGDF}\left(\Phi ,{u}_{1},{u}_{2},{\sigma }_{1}^{2},{\sigma }_{2}^{2}\right)={\int }_{\Omega }{E}_{x}^{LGDF}\left(\Phi ,{u}_{1}\left(x\right),{u}_{2}\left(x\right),{\sigma }_{1}{\left(x\right)}^{2},{\sigma }_{2}{\left(x\right)}^{2}\right){\rm{d}}x.$$

For more accurate computation in evolving the level set function and its evolution, it is necessary to regularize the level set function by penalizing its deviation from a signed distance function, characterized by the following energy functional

16 $$P\left(\Phi \right)=\int \frac{1}{2}{\left(\left|\nabla \Phi \left(x\right)\right|-1\right)}^{2}{\rm{d}}x.$$

As in typical level set methods, it is necessary to regularize the zero level set by penalizing its length to derive a smooth contour during evolution:

17 $$L\left(\Phi \right)=\int \left|\nabla H\left(\Phi \left(x\right)\right)\right|{\rm{d}}x.$$

Therefore, the entire energy function is

18 $$F\left(\Phi ,{u}_{1},{u}_{2},{\sigma }_{1}^{2},{\sigma }_{2}^{2}\right)={E}^{LGDF}\left(\Phi ,{u}_{1},{u}_{2},{\sigma }_{1}^{2},{\sigma }_{2}^{2}\right)+\nu L\left(\Phi \right)+\mu P\left(\Phi \right),$$

where *ν*, *μ* > 0 are weighting constants. In practice, Heaviside function *H* is approximated by a smoothing function *H*_*ε*_ defined by

19 $${H}_{\varepsilon }\left(x\right)=\frac{1}{2}\left[1+\frac{2}{\pi }\arctan \left(\frac{x}{\varepsilon }\right)\right]$$

and the derivative of *H*_*ε*_ is the following smoothing function

20 $${\delta }_{\varepsilon }\left(x\right)={H}_{\varepsilon }^{{\prime} }\left(x\right)=\frac{1}{\pi }\frac{\varepsilon }{{\varepsilon }^{2}+{x}^{2}}.$$

Thus, the energy functional $F\left(\Phi ,{u}_{1},{u}_{2},{\sigma }_{1}^{2},{\sigma }_{2}^{2}\right)$ in Eq. (18) is approximated by

21 $${F}_{\varepsilon }\left(\Phi ,{u}_{1},{u}_{2},{\sigma }_{1}^{2},{\sigma }_{2}^{2}\right)={E}_{\varepsilon }^{LGDF}\left(\Phi ,{u}_{1},{u}_{2},{\sigma }_{1}^{2},{\sigma }_{2}^{2}\right)+\nu {L}_{\varepsilon }\left(\Phi \right)+\mu P\left(\Phi \right).$$

### Gradient Descent Flow

By calculus of variations, it can be shown that the parameters *u*_*i*_ and ${\sigma }_{i}^{2}$ that minimize the energy functional in Eq. (21) satisfy the following Euler–Lagrange equations

22 $$\int \omega \left(y-x\right)\left({u}_{i}\left(x\right)-I\left(y\right)\right){M}_{i,\varepsilon }\left(\varphi \left(y\right)\right){\rm{d}}y=0$$

and

23 $$\int \omega \left(y-x\right)\left({\sigma }_{i}{\left(x\right)}^{2}-{\left({u}_{i}\left(x\right)-I\left(y\right)\right)}^{2}\right){M}_{i,\varepsilon }\left(\varphi \left(y\right)\right){\rm{d}}y=0,$$

where *M*_1,*ε*_(*ϕ*(*y*)) = *H*_*ε*_(*ϕ*(*y*)) and *M*_2,*ε*_(*ϕ*(*y*)) = 1 − *H*_*ε*_(*ϕ*(*y*)). From Eq. (22) and Eq. (23), it is obtained

24 $${u}_{i}\left(x\right)=\frac{\int \omega \left(y-x\right)I\left(y\right){M}_{i,\varepsilon }\left(\varphi \left(y\right)\right){\rm{d}}y}{\int \omega \left(y-x\right){M}_{i,\varepsilon }\left(\varphi \left(y\right)\right){\rm{d}}y}$$

and

25 $${\sigma }_{i}{\left(x\right)}^{2}=\frac{\int \omega \left(y-x\right){\left({u}_{i}\left(x\right)-I\left(y\right)\right)}^{2}{M}_{i,\varepsilon }\left(\varphi \left(y\right)\right){\rm{d}}y}{\int \omega \left(y-x\right){M}_{i,\varepsilon }\left(\varphi \left(y\right)\right){\rm{d}}y}.$$

which minimize the energy functional ${F}_{\varepsilon }\left(\Phi ,{u}_{1},{u}_{2},{\sigma }_{1}^{2},{\sigma }_{2}^{2}\right)$ for a fixed *ϕ*. Minimization of the energy functional *F*_*ε*_ in Eq. (21) concerning *ϕ* can be achieved by solving the gradient descent flow equation

26 $$\frac{\partial \varphi }{\partial t}=-{\delta }_{\varepsilon }\left(\varphi \right)\left({e}_{1}-{e}_{2}\right)+\upsilon {\delta }_{\varepsilon }\left(\varphi \right)div\left(\frac{\nabla \varphi }{| \nabla \varphi | }\right)+\mu \left({\nabla }^{2}\varphi -div\left(\frac{\nabla \varphi }{| \nabla \varphi | }\right)\right),$$

where

27 $${e}_{1}\left(x\right)={\int }_{\Omega }\omega \left(y-x\right)\left[\log \left({\sigma }_{1}\left(y\right)\right)+\frac{{\left({u}_{1}\left(y\right)-I\left(x\right)\right)}^{2}}{2{\sigma }_{1}{\left(y\right)}^{2}}\right]{\rm{d}}y$$

and

28 $${e}_{2}\left(x\right)={\int }_{\Omega }\omega \left(y-x\right)\left[\log \left({\sigma }_{2}\left(y\right)\right)+\frac{{\left({u}_{2}\left(y\right)-I\left(x\right)\right)}^{2}}{2{\sigma }_{2}{\left(y\right)}^{2}}\right]{\rm{d}}y.$$

Note that the image-based term *e*_1_ − *e*_2_ is independent of the scale of local intensities caused by the intensity inhomogeneity. According to the analysis of local intensities, the observed intensities $\overline{I\left(y\right)}$ in *θ*_*x*_ can be approximated by *bI*(*y*), where *b* is the scaling factor. Therefore, the new local intensity means $\overline{\left({u}_{i}\left(x\right)\right.}$ and standard deviations $\overline{\left({\sigma }_{i}\left(x\right)\right.}$ are multiplied by $b:\overline{\left({u}_{i}\left(x\right)\right.}=b\left({u}_{i}\left(x\right)\right.,\overline{\left({\sigma }_{i}\left(x\right)\right.}=b\left({\sigma }_{i}\left(x\right)\right.$, transforming the new image-based terms as $\left(\overline{{e}_{1}}-\overline{{e}_{2}}\right)$

29 $$\begin{array}{lll}(\overline{{e}_{1}}-\overline{{e}_{2}}) & = & {\int }_{\Omega }\omega (y-x)\left[\left.\log (b{\sigma }_{1}(y))+\frac{{(b{u}_{1}(y)-bI(x))}^{2}}{2(b{\sigma }_{1}{(y)}^{2}}\right)\right]{\rm{d}}y\\  &  & -{\int }_{\Omega }\omega (y-x)\left[\left.\log (b{\sigma }_{2}(y))+\frac{{(b{u}_{2}(y)-bI(x))}^{2}}{2(b{\sigma }_{2}{(y)}^{2}}\right)\right]\\ {\rm{d}}y & = & {\int }_{\Omega }\omega (y-x)\left[\left.\log ({\sigma }_{1}(y))+\frac{{({u}_{1}(y)-bI(x))}^{2}}{2({\sigma }_{1}{(y)}^{2}}\right)\right]{\rm{d}}y\\  &  & -{\int }_{\Omega }\omega (y-x)\left[\left.\log ({\sigma }_{2}(y))+\frac{{({u}_{2}(y)-bI(x))}^{2}}{2({\sigma }_{2}{(y)}^{2}}\right)\right]{\rm{d}}y=({e}_{1}-{e}_{2}).\end{array}$$

This property shows that the influence of the image-based term (*e*_1_ − *e*_2_) is invariant to contrast change caused by intensity inhomogeneity.

## Data Availability

The dataset is available under CC0 licensing at Figshare^40^, Kaggle^41^, and GitHub^42^ and it is accompanied by the anonymized patients’ information in xlsx format. For the data analysis and validation, Python version 3.9 was used. The release of the published dataset is 2023-04-04. The possible database updates will always be published with the release date.
